# Prenatal diagnosis, associated findings and postnatal outcome of fetuses with truncus arteriosus communis (TAC)

**DOI:** 10.1007/s00404-021-06067-x

**Published:** 2021-05-24

**Authors:** J. S. Abel, C. Berg, A. Geipel, U. Gembruch, U. Herberg, J. Breuer, K. Brockmeier, I. Gottschalk

**Affiliations:** 1 Division of Prenatal Medicine, Department of Obstetrics and Gynecology, University of Cologne, Kerpenerstr. 34, 50931 Cologne, Germany; 2 Department of Obstetrics and Prenatal Medicine, University of Bonn, Bonn, Germany; 3 Department of Pediatric Cardiology, University of Bonn, Bonn, Germany; 4 Department of Pediatric Cardiology, University of Cologne, Cologne, Germany

**Keywords:** Truncus arteriosus communis, Common arterial trunk, TAC, Aortopulmonary trunk, Congenital heart defect, Fetus, Prenatal diagnosis

## Abstract

**Purpose:**

To assess the spectrum of associated anomalies, the intrauterine course, postnatal outcome and management of fetuses with truncus arteriosus communis (TAC)

**Methods:**

All cases of TAC diagnosed prenatally over a period of 8 years were retrospectively collected in two tertiary referral centers. All additional prenatal findings were assessed and correlated with the outcome. The accuracy of prenatal diagnosis was assessed.

**Results:**

Thirty nine cases of TAC were diagnosed prenatally. Mean gestational age at first diagnosis was 22 weeks (range 13–38). Two cases were lost follow-up. Correct prenatal diagnosis of TAC was made in 87.5% and of TAC subtype in 90.5%. Prenatal diagnosis was incorrect in three cases: one newborn had aortic atresia with ventricular septal defect (VSD) postnatally, one had hypo-plastic right ventricle with dextro transposition of the great arteries (d-TGA) with coarctation of the aorta and a third newborn had tetralogy of fallot (TOF) with abnormal origin of the left pulmonary artery arising from the ascending aorta postnatally. These 3 cases were excluded from further analysis.

In 26.5% of cases, TAC was an isolated finding. 38.2% of fetuses had additional chromosomal anomalies. Among them, microdeletion 22q11.2 was most common with a prevalence of 17.6% in our cohort. Another 3 fetuses were highly suspicious for non-chromosomal genetic syndromes due to their additional extra-cardiac anomalies, but molecular diagnosis could not be provided. Major cardiac and extra-cardiac anomalies occurred in between 8.8% and 58.8%, respectively. Predominantly, extra-cardiac anomalies occurred in association with chromosomal anomalies. Additionally, severe IUGR occurred in 17.6%.

There were 14 terminations of pregnancy (41.2%), 1 (2.9%) intrauterine fetal death, 5 postnatal deaths (14.7%) and 14 (41.2%) infants were alive at last follow-up. Intention-to-treat survival rate was 70%. Mean follow-up among survivors was 42 months (range 6–104). Postoperative health status among survivors was excellent in 78.6%, but 46.2% needed repeated re-interventions due to recurrent pulmonary artery or conduit stenosis. The other 21.4% of survivors were significantly impaired due to non-cardiac problems.

**Conclusion:**

Truncus arteriosus communis is a rare and complex cardiac anomaly that can be diagnosed prenatally with high precision. TAC is frequently associated with chromosomal and extra-cardiac anomalies, leading to a high intrauterine and postnatal loss rate due to terminations and perioperative mortality. Without severe extra-cardiac anomalies, postoperative health status is excellent, independent of the subtype of TAC, but the prevalence of repeated interventions due to recurrent stenosis is high.

## Introduction

Truncus arteriosus communis (TAC) is a rare conotruncal anomaly, representing 1.6% of all newborns with congenital heart disease [1] and 1.07 of 10.000 live births [2, 3]. It is found more commonly in offspring of diabetic mothers [4].

Truncus arteriosus communis is characterized by a single (common) arterial trunk that exits the heart by the way of a single (common) arterial valve and gives rise directly to the coronary, systemic and one or both pulmonary arteries [5, 6]. While aorta and main pulmonary artery (MPA) originate from a common root, failure during the process of separation leads to a persistent common arterial trunk with a common truncal valve with four or more leaflets [7]. This common truncal valve can either be stenotic or insufficient [8]. Frequently, TAC is accompanied by a large ventricular septal defect (VSD) [9] with overriding of the large common arterial trunk.

Two commonly used classifications of TAC describe (1) the different anatomy of the pulmonary arteries which may either arise from a MPA or as direct branches of the aortic arch or the descending aorta and (2) additional aortic arch anomalies. As Collet and Edwards classified the TAC exclusively according to the anatomic origin of the pulmonary arteries and to the spatial relationship between these vessels [10], Van Praagh proposed another anatomical classification which also takes additional aortic arch anomalies into account [11]. As both classification systems have a substantial overlap, we reviewed both classification systems and grouped our cohort into three TAC types according to clinical and surgical aspects and based on both Collet and Edwards and Van Praagh classification.

Because systemic, pulmonary and coronary blood flow are supplied by one common vessel neonates can present with a wide spectrum of clinical features of congestive heart failure depending on the high volume of pulmonary blood flow and the presence or absence of truncal valve insufficiency. As TAC is a cyanotic cardiac anomaly characterized by increased pulmonary blood flow, early surgical repair in the neonatal period may prevent the long-term sequelae of pulmonary over-circulation and heart failure [12–18]. Therefore, precise prenatal echocardiographic diagnosis is mandatory for counselling parents with regard to prognosis and treatment options as well as planning for delivery and postnatal surgical management of the fetuses with TAC.

Indeed, in prenatal situation, it may be difficult to distinguish TAC from other conotruncal malformations namely tetralogy of fallot (TOF) and pulmonal atresia (PA) as both also have a VSD and an overriding aorta.

The components of surgical complete cardiac repair consist of (a) closure of the VSD, (b) right-ventricle-to-pulmonary-artery (RV-PA) conduit and, if necessary, reconstruction of the left ventricular outflow tract in TAC type A4 with hypo-plastic or interrupted aortic arch. Due to pulmonary overflow postnatally, a banding of the pulmonary arteries is frequently performed prior to complete repair. During childhood, re-interventions like dilatation or stenting of the PA or exchange of the RV–PA conduit will frequently be necessary due to recurrent stenoses of PA or insufficiencies of the conduit valve. Additional cardiac and especially extra-cardiac and chromosomal anomalies occur quite frequently and may complicate the surgical course.

The aim of this study was to assess the spectrum of associated cardiac, extra-cardiac and chromosomal anomalies, the intrauterine course and postnatal outcome of fetuses with TAC.

## Methods

All prenatally diagnosed cases of TAC were retrospectively reviewed for intrauterine course and outcome in the perinatal database of two tertiary referral centers for prenatal medicine and fetal echocardiography (University of Cologne and University of Bonn, Germany). All fetuses with TAC were diagnosed between January 2010 and December 2018.

The anatomic survey and fetal echocardiography were performed in a standardized fashion. Fetal echocardiography was carried out by a segmental approach using standardized anatomical planes incorporating pulsed-wave and color Doppler imaging [19, 20]. 5 MHz, 7.5 MHz or 9 MHz sector or curved array-probes were used for all ultrasound examinations (ATL HDI 5000 and IU22 Philips, Hamburg, Germany; Voluson 730 Pro and Expert, E8 and E10, respectively, GE Healthcare, Solingen, Germany). A pediatric cardiologist attended at least one of the prenatal ultrasound examinations and subsequently counselled the patients. Following delivery and initial care by the attending neonatologist, all newborns were examined by a pediatric cardiologist within 12 h after birth. Conventional karyotyping was performed in all cases, predominantly prenatally, otherwise postnatally.

Prenatally, we distinguished three different TAC types based on both Collet and Edwards’ and Van Praagh's classification, as both classification systems have a substantial overlap and both were used inconsistently in pediatric and cardio-surgical literature:

- **Type 1** corresponds to both Collet and Edwards' *and* Van Praagh's type 1 and included all cases of TAC with an existing MPA.
- **Type 2** combined Collet and Edwards' type 2 and 3, which corresponds to Van Praagh's type 2 and included all TAC with a separate branching of the pulmonary arteries, either close together or at some distance from each other, as prenatal differentiation between both Collet and Edwards' types is barely possible.
- **Type A4** corresponds to Van Praagh's type A4 and included all TAC with aortic arch anomalies including hypo-plastic or interrupted aortic arch.

Additionally, we described any functional abnormality of the truncal valve as dysplastic, stenotic or insufficient, any major cardiac and extra-cardiac anomalies as well as any chromosomal or non-chromosomal syndromic anomalies. Minor anomalies like right aortic arch, persistent left superior vena cava, aberrant right subclavian artery and single umbilical artery were documented, but not classified as associated cardiac or extra-cardiac anomalies, respectively.

All cases were classified according to pregnancy outcome into five groups: termination of pregnancy (TOP), intrauterine fetal (IUFD) or neonatal death (NND), death in infancy or childhood (CHD) and survivors. Neonatal death was defined as death within the first 28 days of live, CHD as any death after 28 days of live. Postnatal medical files of echocardiography, cardiac catheterization, surgery or autopsy were available for confirmation of the prenatal diagnosis in all live born children. Pre and postnatal diagnoses of all 22 live born children and 2 terminated pregnancies with post-mortal autopsy were compared to assess the accuracy of prenatal diagnosis.

All data were retrieved from medical files, stored ultrasound images and, if available, from ultrasound video recordings. The following variables were assessed, as far as retrospectively achievable: maternal age, gestational age at first diagnosis, type of TAC, associated cardiac, extra-cardiac and chromosomal or non-chromosomal genetic anomalies.

Pregnancies with a postnatal diagnosis differing from TAC were excluded from further outcome analysis. Only for assessment of accuracy of prenatal diagnosis, these cases were included. Both cases, that were lost to follow-up, were also excluded from the study.

Intergroup comparisons were made using the chi-square test, Student’s *t* test or Fisher´s exact test when appropriate. Values are given as mean ± SD unless otherwise indicated. *P* < 0.05 was considered statistically significant. This retrospective study was approved by the local ethical committee of human research (No 20-1517).

## Results

**Fig. 1:**
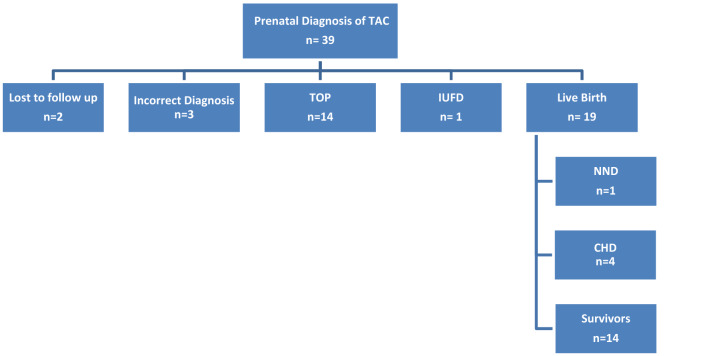
Outcome of 39 cases with prenatally diagnosed TAC. *TOP* termination of pregnancy, *IUFD* intrauterine fetal death, *NND* neonatal death, *CHD* death in infancy or childhood

Truncus arteriosus communis was diagnosed in 39 fetuses during the study period (Fig. 1). Mean gestational age at first diagnosis was 22 weeks (range 13–38). In 8 cases diagnosis was made before 20 weeks of gestation.

Two cases were lost to follow-up and were excluded from further outcome analysis. Another 3 cases had an incorrect prenatal diagnosis and were also excluded from outcome analysis, but included for assessment of diagnostic accuracy. In the first case, initial prenatal echocardiography was performed not before 38 weeks of gestation and maternal body mass index (BMI) was 39. Postoperative diagnosis was Aortic Atresia with VSD instead of TAC. In the second case, first echocardiography was performed at 34 weeks and maternal BMI was 37. Postnatal diagnosis was Hypo-plastic Right Ventricle with d-TGA and Coarctation of Aorta. In the third case, the pregnant woman was only seen once at early 19 weeks and prenatal diagnosis of TAC type 1 was postnatally reclassified as Tetralogy of Fallot with abnormal origin of the left pulmonary artery arising from the ascending aorta.

The main reasons for referral in one of our two participating referral centers were either (a) suspicion of congenital heart disease due to an abnormal three vessel view or (b) presence of extra-cardiac anomalies in externally performed ultrasound in between 41% and 44%, respectively. In the latter, diagnosis of TAC was then made as an additional finding. In 15% of cases, diagnosis was made as (c) an incidental finding during routine first or second trimester anomaly scan in our two centers. Despite consistent improvement in fetal echocardiography during the last decade, there was no tendency towards an earlier diagnosis during the 8-year study period in our cohort.

### Additional genetic, cardiac and extra-cardiac anomalies

After exclusion of cases with incorrect diagnosis and both cases that were lost to follow-up, 9 of our 34 fetuses (26.5%) had isolated TAC. Chromosomal anomalies occurred in 13 fetuses with a prevalence of 38.2% (Table 1). Among them, microdeletion 22q11 was most common with a prevalence of 17.6% in our cohort. Due to their complex additional anomalies, another 3 fetuses were highly suspicious for non-chromosomal genetic syndromes, but molecular diagnosis could not be provided. Invasive prenatal testing was performed in 25 pregnancies. Postnatally, none of the remaining newborns had additional chromosomal anomalies.

**Table 1:** All 34 cases with TAC according to additional anomalies and outcome

No	Karyotype	Cardiac anomalies	Extra-cardiac anomalies	Outcome
1	Microdeletion 22q11	ARSA	Thymus aplasia	TOP
2	Microdeletion 22q11	ARSA	Thymus aplasia	TOP
3	Microdeletion 22q11	–	Hygroma colli	TOP
4	Microdeletion 22q11	–	Thymus aplasia, cerebellar hypoplasia, microcephaly, SUA	CHD
5	Microdeletion 22q11	–	Thymus aplasia, IUGR, SUA	CHD
6	Microdeletion 22q11	–	Thymus aplasia, SUA	Survivor
7	46, XY del(1) (q42.1)	–	Corpus callosum agenesis, Dandy-Walker malformation, retrognathia, SUA	TOP
8	46, XY del(2) (q31q32.2)	DORV, HLV	Hypertelorisma, syndactylia	TOP
9	46, XX del(9q34)	RAA, LPSVC	Thymus aplasia, cerebellar hypoplasia, microcephaly, SUA	TOP
10	46, XX der9	–	–	TOP
11	Trisomy 9	–	Myelomeningocele	TOP
12	Trisomy 13	HLV	Holoprosencephaly, proboscis, hypoterlorism, polydactylia both hands and feet	TOP
13	Trisomy 16 Mosaicism	–	Severe early IUGR	TOP
14	46, XX*	–	Uretro-pelvic junction obstruction, gall bladder aplasia, severe IUGR, SUA	TOP
15	46, XY*	–	Holoprosencephaly, medial lip and cleft palate, unilateral anophthalmia	TOP
16	46, XX*	RAA	Retrognathia, cleft palate, clenched fingers, polydactyly, kyphoscoliosis, micromelia	Survivor
17	46, XX	–	Holoprosencephaly, hypotelorism, microcephaly	TOP
18	46, XY	–	Holoprosencephaly, severe IUGR	IUFD
19	46, XY	–	Syringomyelia	Survivor
20	46, XY	RAA	Moderate hydronephrosis	Survivor
21	46, XX	–	Retrognathia, cleft palate	Survivor
22	46, XX	LPSVC, RAA	Left-sided diaphragmatic hernia, SUA	Survivor
23	46, XY	–	Severe IUGR	CHD
24	46, XX	–	Severe IUGR	Survivor
25	46, XY	–	SUA	TOP
26	46, XY	RAA	SUA	NND
27	46, XY	AVSD, LPSVC, RAA	SUA	Survivor
28	46, XX	–	–	CHD
29	46, XX	–	–	Survivor
30	46, XX	–	–	Survivor
31	46, XX	–	–	Survivor
32	46, XY	–	–	Survivor
33	46, XY	–	–	Survivor
34	46, XY	–	–	Survivor

*ARSA* aberrant right subclavian artery, *RAA* right aortic arch, *LPSVC* left persistent superior vena cava, *DORV* double-outlet right ventricle, *HLV* hypo-plastic left ventricle, *AVSD* atrio-ventricular septal defect, *IUGR* intrauterine growth restriction, *SUA* singular umbilical artery

*Highly suspicious for non-chromosomal genetic syndrome

Additional major cardiac anomalies occurred only in 3 (8.8%) cases and included hypo-plastic left ventricle, double-outlet right ventricle and atrio-ventricular septal defect (Table 1). Hypo-plastic or interrupted aortic arch was not described as additional cardiac defect, as it was classified as TAC type A4. Minor cardiac anomalies were right aortic arch (RAA), persistent left superior vena cava (LPSVC) and aberrant right subclavian artery (ARSA) in 6 (17.6%), 4 (11.7%) and 2 (5.9%) cases, respectively.

Major extra-cardiac anomalies occurred in 20 (58.8%) cases, including cerebellar hypoplasia, Dandy-Walker-Malformation, holoprosencephaly, facial clefts, myelomeningocele or syringomyelia, diaphragmatic hernia and corpus callosum agenesis (Table 1). Frequently, those anomalies were associated with additional chromosomal anomalies or with fetuses that were highly suspicious for non-chromosomal genetic syndromes. Severe IUGR occurred in another 6 (17.6%) cases. Singular umbilical artery was classified as minor anomaly and was seen in 11 (32.4%) fetuses.

The high prevalence of additional genetic and major extra-cardiac anomalies led to a high pre- and postnatal loss rates, mainly due to terminations of pregnancy and spontaneous demise in the early childhood, respectively.

### Outcome

Termination of pregnancy (TOP) was opted in 14 of our 34 (41.2%) cases (Fig. 1). All but one fetus had additional major anomalies including 10 fetuses with chromosomal anomalies.

Intrauterine fetal death (IUFD) at 21 weeks of gestation occurred in 1 fetus (2.9%) with alobar holoprosencephaly and severe early intrauterine growth restriction (IUGR).

Nineteen (55.9%) neonates with postnatally confirmed TAC were born alive. One (2.9%) neonate died during neonatal period (NND) and 4 (11.8%) infants in early childhood (CHD). The neonate had isolated TAC and died 5 days after completed cardiac repair due to multiorgan failure while he was treated with extra corporal membrane oxygenation (ECMO). The remaining 4 infants died within their first 3 months of life: one infant had microdeletion 22q11 and died after pulmonal arterial (PA) banding due to intraventricular hemorrhage and right heart and renal failure. A 2nd infant with microdeletion 22q11 died after completed cardiac repair due to heart and renal failure while he was treated with a pacemaker and peritoneal dialyses simultaneously. A 3rd infant who was delivered severely growth restricted at 32nd week of gestation (birth weight 880 g), underwent PA-banding and stenting of the persistent arterial duct and died at the age of 3 months due to prematurity and heart failure. The 4th infant developed necrotizing enterocolitis postnatally and underwent placement of a colostomy on her 10th day of life. During surgery, she had myocardial infarction with hypoxic brain damage and developed severe renal failure. She died at the age of 7 weeks after her parents opted for palliative care.

Fourteen children with confirmed TAC were alive at last follow-up, resulting in an overall survival rate of 41.2%. After exclusion of TOP, the intention-to-treat survival rate was 70%. Mean follow-up among survivors was 42 months (range 6–104).

### Postnatal cardiac surgery

All survivors underwent cardio-surgical treatment. Due to initial pulmonary overflow postnatally, 11 of 19 (57.9%) neonates needed initial banding of the pulmonary arteries prior to complete cardiac repair, 5 (26.3%) of them received additional stenting of the arterial duct. Single-stage complete cardiac repair with patch-closure of the VSD and insertion of a right-ventricle-to-pulmonary-artery (RV–PA) bovine conduit were performed in 11 (78.6%) infants, predominantly within their first weeks of life. Among them, 6 neonates needed additional reconstruction of their hypo-plastic or interrupted aortic arch. Another 2 (14.3%) infants with TAC type 1 suffered from progressive cyanosis after conduit insertion due to recurrent conduit stenosis and had to undergo additional Blalock–Taussig shunting and subsequent Glenn anastomosis. One (7.1%) infant only had stenting of persistent arterial duct so far and is still awaiting complete repair.

After cardiac surgery, recurrent stenoses of the conduits or pulmonary arteries (PA) as well as insufficiencies of the valved conduits occurred frequently during childhood. Six (46.2%) of the 13 infants who had achieved complete cardiac repair required 2–6 re-interventions (mean 3.3), either stenting or dilatation of PA. Among them, 2 (15.4%) infants required additional conduit-exchange. Another infant (7.7%) developed complete heart block and achieved a pacemaker.

Postoperative health status among our survivors was either excellent or significantly impaired. Eleven (78.6%) infants had an excellent health status at last follow-up, although one of them reported on mildest cyanosis during intense physical activity and another one on first-grade heart insufficiency during intense activity. Indeed, the remaining 3 (21.4%) infants are significantly impaired due to non-cardiac problems: one infant had retrognathia with cleft palate and suffered from dysphagia and impaired hearing. Another infant was affected by sepsis and necrotizing enterocolitis, needed tracheostoma for intermittent home ventilation and respiration therapy due to total atelectasis and severely suffered from seizure, psychomotor and mental retardation. The third, presumably syndromal infant with multiple extra-cardiac anomalies needed tracheostoma and suffered, among other problems, from severe pulmonary hypoplasia, hypertension and impaired hearing.

### TAC typing and accuracy of prenatal ultrasound

**Fig. 2:**
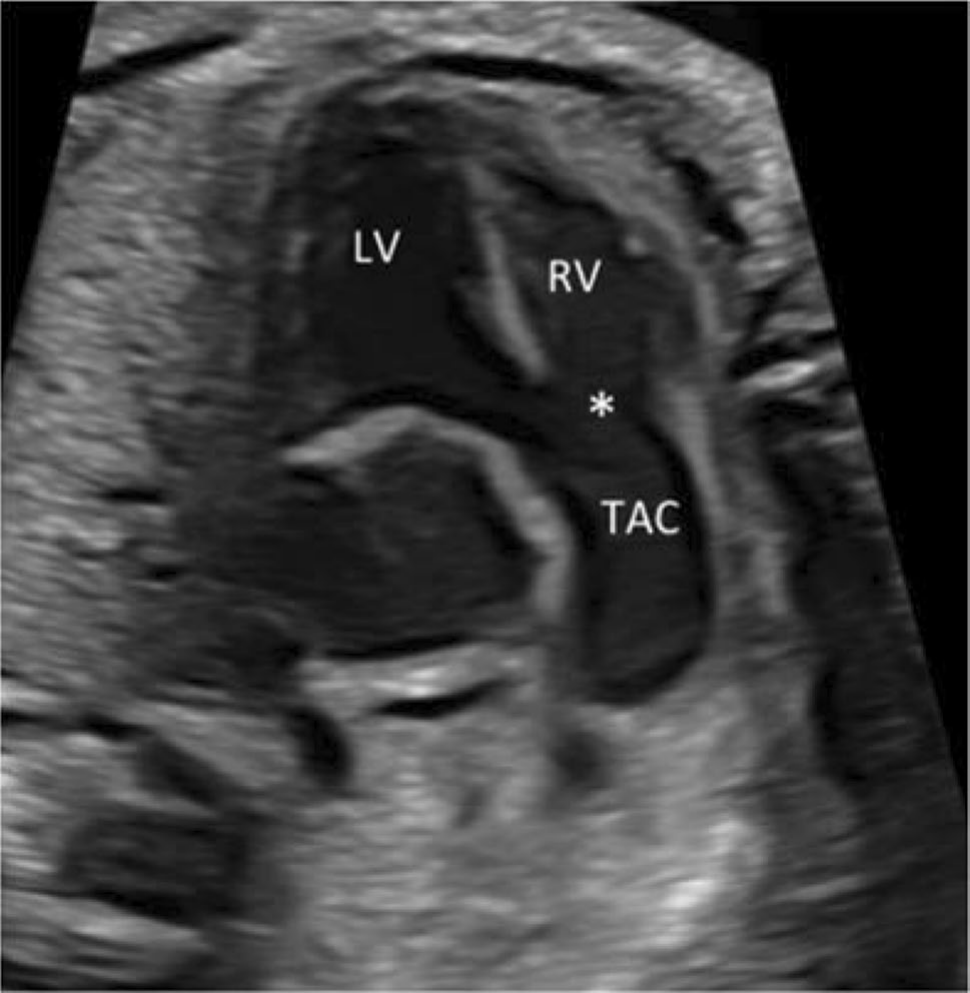
Common arterial trunk (TAC) overriding a large VSD (*)

**Fig. 3:**
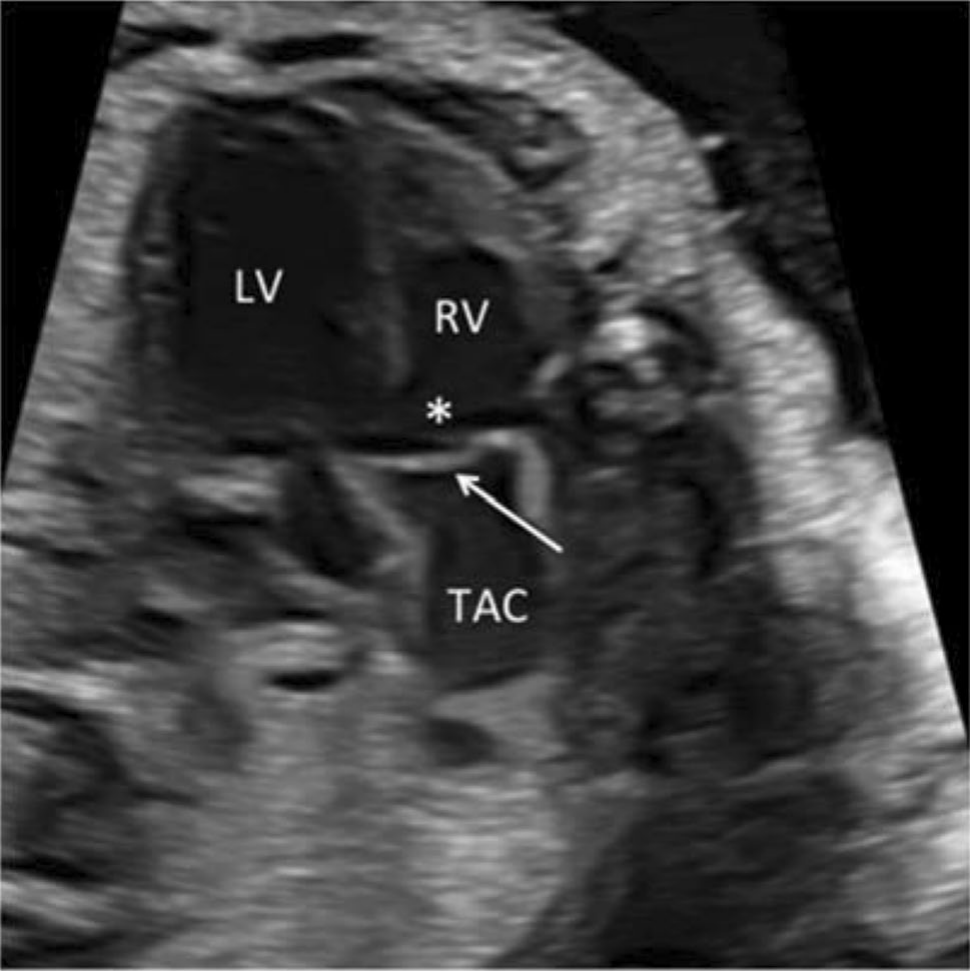
Large common arterial trunk valve (arrow). *LV* left ventricle, *RV* right ventricle, *VSD

**Fig. 4:**
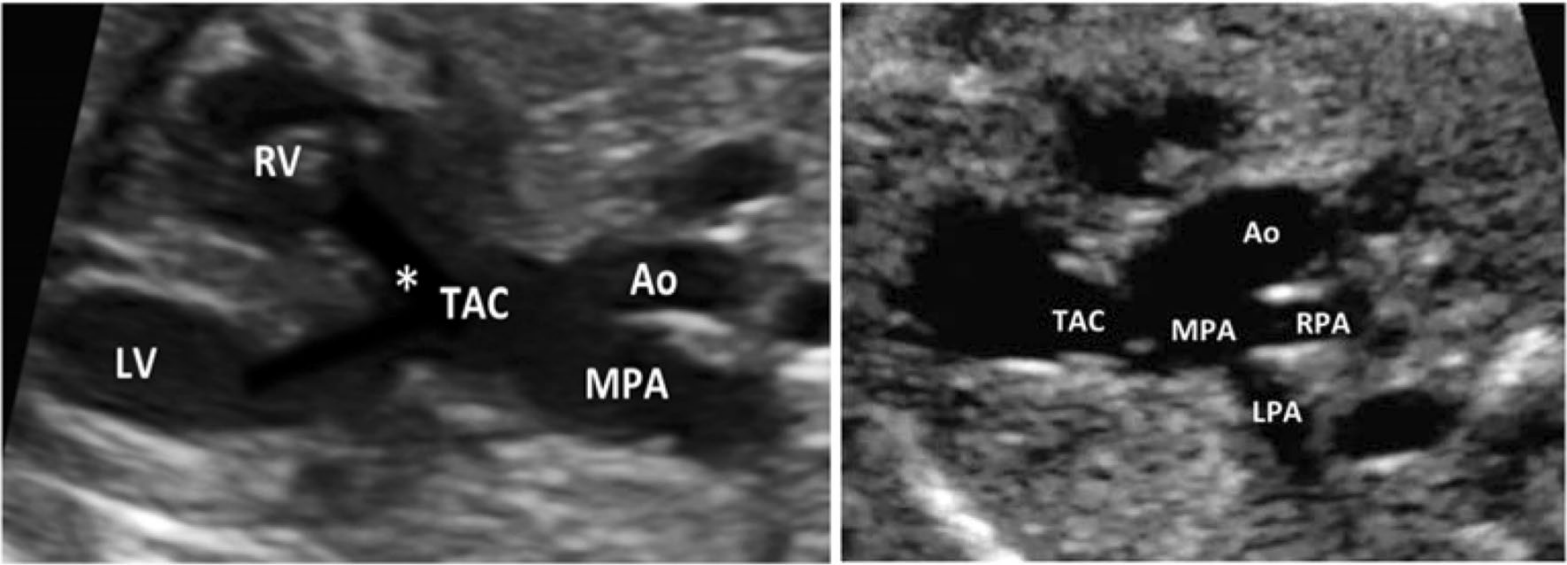
TAC type 1 with overriding common arterial trunk (TAC). The aorta (Ao) arises from the right side of the common trunk, a short main pulmonary artery (MPA) from the left side of the common trunk. The MPA then divides into the right (RPA) and left pulmonary artery (LPA). (*VSD; *RV* right ventricle, *LV* left ventricle)

**Fig. 5:**
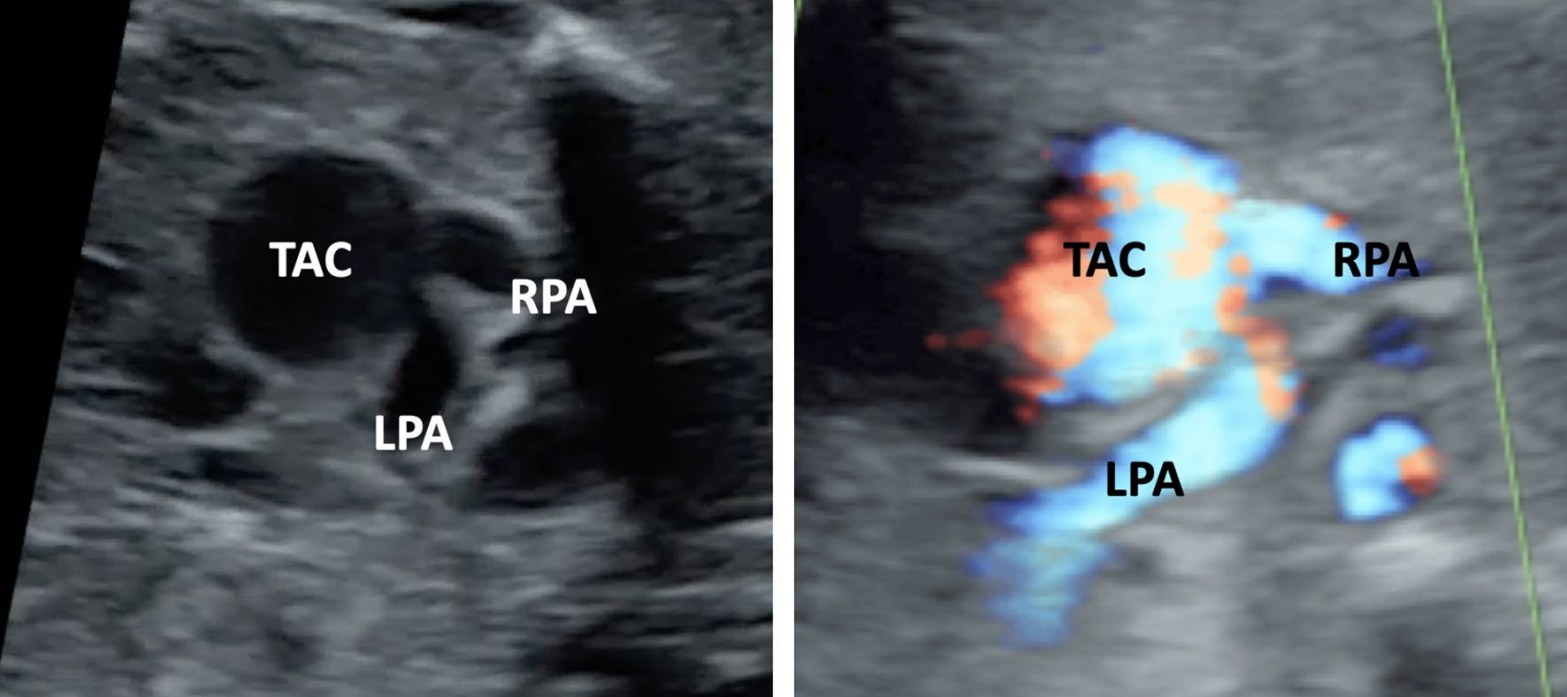
TAC type 2. Cross-section of the common arterial trunk (TAC) with a separate branching of the right (RPA) and left pulmonary artery (LPA). In this case the branching of both pulmonary arteries is close together

**Fig. 6:**
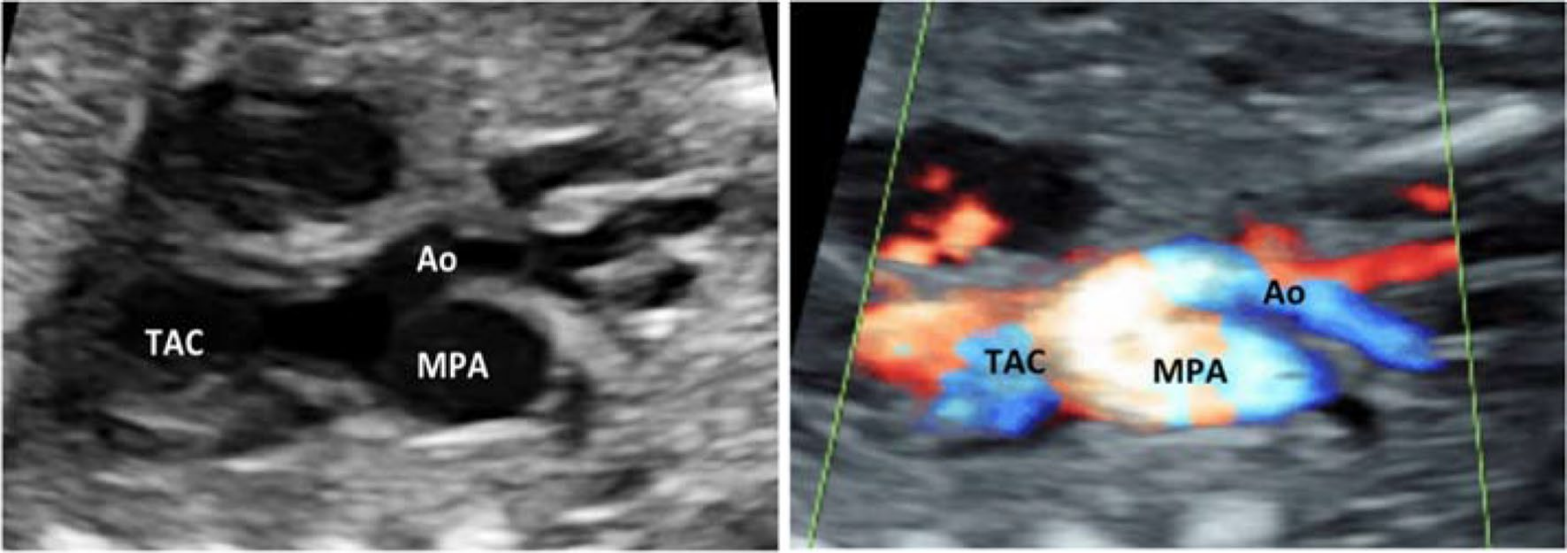
TAC type A4. The aorta (Ao) and the main pulmonary artery (MPA) arise from the common arterial trunk (TAC). The interrupted aorta divides into the brachiocephalic and left common carotid arteries (interrupted aortic arch type B)

To determine the TAC types, we included all cases with confirmed diagnosis of TAC, either by post-mortal autopsy (*n* = 2) or postnatal echocardiography and cardiac surgery (*n* = 19). The most common type was type **1** in 38.1%, followed by type **A4** in 33.3% and type **2** in 28.6% (Figs. 2, 3, 4, 5 and 6). In 23.5%, the common trunk valve was severely insufficient and/or stenotic.

To determine the **accuracy** of prenatal diagnosis, we included all cases with prenatally presumed diagnosis of TAC and confirmed postnatal diagnosis of congenital heart defect, either post-mortally by autopsy (*n* = 2) or postnatally by echocardiography (*n* = 22). Correct prenatal diagnosis of TAC was made in 21 (87.5%) cases. Among them, exact TAC subtyping was correct in 19 (90.5%) cases.

## Discussion

Truncus arteriosus communis is a rare conotruncal anomaly which accounts for 1–2% of all congenital heart defects in newborns [1]. Prenatal incidence is higher due to a significantly high prenatal loss rate [21]. As reported in literature [11, 22], both sexes in our cohort were equally affected with 52.4% of affected male and 47.6% of affected female fetuses.

Due to the rarity of the disease, previously published studies on prenatally diagnosed TAC described either extremely small cohorts with only very short postnatal follow-up periods of less than a year [21, 23–25] or included prenatally as well as postnatally diagnosed cases of TAC without any information about additional anomalies and type of surgical treatment [26]. In addition, the numbers of survivors in those series were extremely small, due to the high prevalence of terminations of pregnancy, intrauterine losses and considerable pre and post-surgical mortalities. Therefore, most information concerning the outcome of newborns with TAC is available from pediatric series only. Our current study is the largest cohort of exclusively prenatally diagnosed TAC with a larger number of survivors and significantly longer follow-up period with known surgical treatment and post-surgical health status and therefore may improve the quality of prenatal counselling of affected couples.

Anomalous migration of neural crest cells through the branchial arch vessels during cardiogenesis leads to an incomplete septation (persistence) of the truncus arteriosus (the distal portion of the cardiac outflow tract of the embryonic heart tube) into the pulmonary artery and the aorta [5, 7]. Defective separation affects the aortic sac, the ventriculoarterial junction and the outlet [27]. Consequently, the morphologic characteristics of TAC are (1) the common arterial duct itself, (2) a common arterial orifice and valve and (3) a ventricular septal defect (VSD) in the outflow region [5, 9, 27]. The VSD is usually large and located in the infundibular region, but this defect can also be absent or very small [9, 11, 28–31]. The common arterial valve is often malformed, stenotic or incompetent, the ductus arteriosus is small or absent [8, 11, 32]. In our cohort, a VSD was present in all cases and the common truncal valve was stenotic or insufficient in 23% of cases.

### Classification of TAC and impact on outcome

The classification by Collet and Edwards [10] describes 4 types of TAC according to the anatomic origin of and spatial relationship between the pulmonary arteries. In type I, a short MPA arises from the left side of the common trunk and divides into the right (RPA) and left pulmonary artery (LPA). In types 2 and 3, both pulmonary arteries arise separately from the posterior part of the common trunk, either close to each other (type 2) or at some distance from each other (type 3). In type 4, the pulmonary arteries arise from the aortic arch or descending aorta as direct branches. Indeed, type 4 is no longer considered a variant of TAC but is rather classified as as pulmonary atresia with VSD and aortopulmonary collateral arteries (PAVSD + MAPCAs).

As aortic arch anomalies including right aortic arch, hypo-plastic or interrupted aortic arch, occur quite frequently in TAC [33–37], Van Praagh proposed another classification [11]: Van Praagh’s type A1 is similar to Collet and Edwards’ type 1. Van Praagh's type A2 combines Collet and Edwards' type 2 and type 3. As exact distinction between type A1 and A2 may be difficult even with angiographic evaluation, it has been proposed that both types should be merged into one group [22]. Van Praagh's type A3 describes a unilateral atresia of the pulmonary arteries and pulmonary blood supply through ductal or aortopulmonary collateral vessels. Van Praagh's type A4 refers to aortic arch anomalies and describes TAC with interrupted aortic arch.

In accordance with the Society of Thoracic Surgeons, the Congenital Heart Surgery Database Committee [38] and the European Association for Cardiothoracic Surgery, we grouped our cohort into three TAC types: Type 1 (Collet and Edwards), type 2 (Collet and Edwards) and A4 (Van Praagh).

Data concerning the prevalence of different TAC subtypes in prenatal series are rare. The only published prenatal series with subtype classification by Lee et al. included only 8 cases with confirmed postnatal diagnosis [23]. Due to their small cohort, no reliable information about the prevalence of different subtypes can be obtained. In pediatric series, type 1 is considered the most common type with a prevalence of 47–50%, followed by type 2 in 21% and type A4 in only 2–12% of cases [22, 39]. In accordance, type 1 was the most common type in our cohort. But in contrast, type A4 was much more common in our series with a prevalence of 33%. The significant higher prevalence of type A4 in our prenatal series cannot be explained conclusively, as intrauterine mortality was not higher in fetuses with TAC type A4 compared with the remaining types.

Also, several pediatric series stated that TAC type A4 has a worse outcome compared to the remaining types. Miyamoto et al. stated that overall perioperative mortality in infants with TAC with IAA were indeed decreasing in recent decades, but still high [39]. Perioperative mortality in their own series including 10 infants with TAC type A4 was 50%. Konstantinovi et al. confirmed the rather worse prognosis of infants with TAC type A4 with a 10-year survival rate of not more than 31% [33]. In contrast to those pediatric series, we could not confirm the increased postnatal mortality or morbidity in TAC type A4 after prenatal diagnosis: postoperative survival rate at last follow-up did not differ significantly in TAC with (71.3%) or without IAA (72.7%). Furthermore, postoperative health status was comparably good in both groups: 80.0% of infants with TAC with IAA and 78.7% of infants with TAC without IAA lived without any limitations after surgery. We could state that after prenatal diagnosis of TAC, prognosis is good independently of the subtype, as the subtype did not influence mortality or morbidity in our survivors. If the health status was impaired postnatally than exclusively, it was because of additional major non-cardiac or chromosomal anomalies.

### Accuracy of prenatal diagnosis and impact on Outcome

Precise prenatal diagnosis of TAC can be achieved in the majority of fetuses. Previously published studies report on overall accuracy of prenatal diagnosis between 71% [23] and 87% [24, 40] (Table 2). Our study confirms that TAC can be diagnosed prenatally with high accuracy. The correct diagnosis of TAC was made in 87%, but diagnosis was usually not made prior to 21 weeks of gestation, presumably due to a predominantly normal looking four-chamber view that conceals this defect at basic cardiac screening. Reduced visibility at echocardiography in very early or late gestational age and additional maternal obesity were negative factors contributing to incorrect diagnosis: in our three cases with incorrect diagnosis, two obese patients were initially referred to our institution for initial echocardiography not before 34 and 37 weeks of gestation, respectively. The third patient was referred for echocardiography only once at early 18 weeks of gestation. Correct subtyping of TAC types 1, 2 or A4 could be achieved in more than 90% in our cohort. Despite consistent improvement in fetal echocardiography during the last decades, there was no tendency towards an earlier diagnosis during the 8-year study period in our cohort.

**Table 2:** Meta-analysis of the current and previously published series

Author	Current study	Swanson [26]	Lee [23]	Volpe [21]	Duke [24]	Gomez [25]
Included cases (*n* =)	34	43	12	23	17	10
Diagnosis pre or postnatally	Pre	Pre + post	Pre	Pre	Pre	Pre
TOP (*n* =)	14 (41%)	17 (40%)	4 (33%)	8 (35%)	4 (24%)	9 (90%)
IUFD (*n* =)	1 (2.9%)	2 (4.7%)	0	2 9%	0	0
Life Birth (*n* =)	19 (55.8%)	24 (56%)	8 (66%)	13 (57%)	13 (76.5%)	1 (10%)
NND/CHD (*n* =)	5 (14.8%)	8 (18.6%)	2 (17%)	5 (22%)	8	–
Overall Survival (*n* =)	14/34 (41%)	16/43 (37%)	6/12 (50%)	8/23 (35%)	5/17 (29.4)	1/10 (10%)
Intention to treat survival (*n* =)	14/20 (70%)	16/26 (62%)	6/8 (75%)	8/15 (53%)	5/12 (42%)	1/1 (100%)
Microdeletion 22q11.2 (*n* =)	6/34 (17.6%)	5/17 (29%)	0	6/19 (32%)	0	1/10 (10%)
Accuracy of prenatal diagnosis	87.5%	79%	71%	96%	87%	66.7%
Peri or post-surgical mortality (*n* =)	5/19 (26%)	4/17 (24%)	2/8 (25%)	2/8 (25%)	2/8 (25%)	No data
Major associated	74%					
Anomalies	38% chromos 9% cardiac 59% extra-cardiac	No data	50% cardiac 17% extra-cardiac	35% cardiac 43% extra-cardiac	No data	40% chrom 27% extra-cardiac
TAC type	38% type 1 29% type 2 33% type A4	No data	50% type 1 42% type 2 8% type A4	No data	No data	No data
Surgery	*n* = 14 13 complete repair 1 awaiting repair	No data	No data	*n* = 8 6 complete repair 2 palliative treatm	*n* = 8 7 repair 1 palliative	No data
Follow-up (months)	42	No data	No data	10	41	10

The importance of an accurate prenatal diagnosis in counselling parents with regard to prognosis and in predicting the type of postnatal surgical approach is obvious. Swanson et al. showed that infants with prenatal diagnosis of TAC had significantly earlier surgical intervention than infants with postnatal diagnosis [26]. Early surgical intervention might decrease mortality and morbidity as it prevents the long-term sequelae of pulmonary over-circulation and heart failure [17, 18]. In addition, prenatal diagnosis allows for optimized planning of delivery and postnatal surgical treatment [26].

### Additional anomalies and impact on prognosis

In our cohort, the prognosis of fetuses with TAC mainly depended on the severity of additional extra-cardiac and chromosomal anomalies rather than on the cardiac defect itself. Pediatric series reported on a high prevalence of additional cardiac anomalies including absence of the ductus arteriosus in 50% of cases and coronary artery anomalies in more than a third of cases [34–36, 41]. In our cohort, additional major cardiac anomalies were rarely seen in only 9% of cases. As the precise assessment of coronary anomalies is extremely challenging in prenatal situation, those anomalies may escape prenatal diagnosis in many cases.

In contrast, chromosomal and extra-cardiac anomalies occurred in a significant proportion of our fetuses with a prevalence of 38.2% and 58.8%, respectively. Only 26% of our fetuses had isolated TAC. This is in accordance with current literature, describing additional anomalies in 40–78%, even though in extremely small cohorts only [21, 23, 25, 42, 43] (Table 2). Microdeletion 22q11 was the most common chromosomal anomaly with a prevalence of 17.6% in our cohort and a prevalence of 10–32% in other series [21, 25, 26, 42, 43]. In contrast, major extra-cardiac anomalies occurred more frequently in our cohort with a prevalence of 58.8%, compared to 17–43% in other series [21, 23, 25]. This discrepancy might either be explained by the extremely small cohorts in all other prenatal series, or those series included no data on the presence and severity of additional anomalies, type of surgery or health status at follow-up examinations [21, 23–26]. As comparison with other prenatal series is hardly possible, larger series would be advantageous to confirm our data, as those anomalies significantly complicate the surgical course, contribute to the postnatal mortality and morbidity and may influence parents' decision to continue or terminate the pregnancy. Although, larger series will be hardly achievable due to the rarity of this cardiac anomaly. Certainly, prenatal diagnosis of TAC should trigger a meticulous search for additional anomalies and karyotyping should be offered to all parents.

The prevalence of terminations of pregnancies in previously published series varies considerably between 24% [24], 40% [26] and 75% [25]. In our cohort, 41% of pregnancies were terminated. The low prevalence of TOP in the cohort by Duke et al. [24] might be due to the fact that diagnosis was made rather late in pregnancy and beyond the time period, in which TOP would have been a legal option. In contrast, the high termination rate of 75% in the cohort by Gomez is astonishing as more than half of the terminated pregnancies had isolated TAC with a presumably good prognosis. In addition, Gomez's series included only 8 cases with an accuracy of prenatal diagnosis of only 67% [24].

### Surgical outcome

Although TAC is considered a cyanotic congenital heart defect characterized by increased pulmonary blood flow, cyanosis is not a constant feature in neonatal period [12]. However, if TAC is left untreated, the increasing amount of mixed blood perfusing the pulmonary circulation leads to an increase in pulmonary vascular resistance and to cardiac heart failure [44]. Due to pulmonary overflow, 58% of our neonates needed initial banding of the PA prior to their complete cardiac repair. Among them, 26% needed additional stenting of the arterial duct.

Single-stage complete cardiac repair was performed in 78% of our infants, predominantly within their first weeks of life. Among them, half of those infants needed additional reconstruction of their hypo-plastic or interrupted aortic arch. As 14% of infants with TAC type 1 suffered from progressive cyanosis after conduit insertion due to recurrent conduit-stenoses, those infants needed to undergo additional Blalock–Taussig shunting and subsequent Glenn anastomosis.

In contrast to current literature, postoperative health status among our survivors was either excellent in 78% of infants or significantly impaired in 22%. All individual limitations in health status could exclusively be attributed to additional extra-cardiac or genetic anomalies, e.g. dysphagia and impaired hearing due to severe retrognathia.

Surgical re-interventions after cardiac repair were common during follow-up. In our cohort, 46.2% of all infants had to undergo 2–6 re-interventions (mean 3.3). Unfortunately, no data on prevalence or frequency of re-interventions were included in other prenatal series, but postnatal pediatric series confirm the high prevalence of re-interventions of 75% during 10-year follow-up [45].

In conclusion, the prognosis and postoperative health status are excellent in absence of severe extra-cardiac or genetic anomalies. The prognosis is good, independently of the type of TAC, the presence of additional aortic arch anomalies and the competence of the common truncal valve, but the prevalence of repeated interventions due to recurrent stenoses is high.

### Meta-analysis of literature

#### Limitations

Our study also has a number of limitations. Although our series is one of the largest cohorts with exclusively prenatal cases with the longest follow-up period, the number of newborns who underwent surgery is still small due to the rarity of the cardiac defect itself and the high pre- and postnatal loss rates. The size of our cohort did not allow for identifying any additional predictors of outcome. Although median follow-up was 42 months in our cohort, no data on long-term outcome could be achieved so far. A further limitation is its retrospective design, that limits the assessment of detailed spatial relationship of some cardiac strutures.
